# Membrane Treatment to Improve Water Recycling in an Italian Textile District

**DOI:** 10.3390/membranes15010018

**Published:** 2025-01-09

**Authors:** Francesca Tuci, Michele Allocca, Donatella Fibbi, Daniele Daddi, Riccardo Gori

**Affiliations:** 1 Department of Civil and Environmental Engineering, University of Florence, Via di Santa Marta 3, 50139 Firenze, Italy; riccardo.gori@unifi.it; 2 Gestione Impianti Depurazione Acque S.p.A., Via Baciacavallo 36, 59100 Prato, Italy; m.allocca@gida-spa.it (M.A.); d.fibbi@gida-spa.it (D.F.); d.daddi@gida-spa.it (D.D.)

**Keywords:** textile wastewater, membrane treatment, nanofiltration, hardness removal, resource recovery, textile industry sustainability

## Abstract

The textile district of Prato (Italy) has developed a wastewater recycling system of considerable scale. The reclaimed wastewater is characterized by high levels of hardness (32 °F on average), which precludes its direct reuse in numerous wet textile processes (e.g., textile dyeing). Consequently, these companies utilize ion exchange resins for water softening. However, the regeneration of the resins results in an increased concentration of chlorides in the reclaimed wastewater that exceeds the limit set by Italian regulations for the reuse of water for irrigation purposes. The objective of this study is to investigate the potential of membrane filtration as an alternative method for removing hardness from water. Therefore, an industrial-scale ultrafiltration-nanofiltration (UF-NF) pilot plant was installed to test the rejection of hardness from the reclaimed wastewater. The experiment employed two types of NF membranes and three permeate fluxes (27, 35, and 38 L·m^−2^·h^−1^) for testing. The results demonstrated that the system could remove hardness with efficiencies exceeding 98% under all conditions tested. The experimental findings indicate that the UF-NF system has the potential to be employed as a post-treatment step to render the reclaimed wastewater suitable for all textile finishing processes and to expand the scope for reuse.

## 1. Introduction

It is estimated that global water demand will increase annually. Estimates indicate that by 2030, water requirements will reach approximately 6900 billion m^3^, representing a 64% increase over the water accessible to most nations [1]. Furthermore, industrial withdrawals are expected to account for 22% of global demand in 2030 [2]. The textile industry is among the most water-intensive industrial sectors, with a water consumption rate of 200–400 L of freshwater per kg of finished product and a wastewater discharge volume comprising up to 70% of total freshwater consumption [3]. Clothing production is estimated to reach 160 million t by 2050, which is three times the current level. Consequently, the consumption of water and energy will increase [4]. In this context, water recycling and reuse have become a primary strategy for conserving water resources.

**Figure 1:**
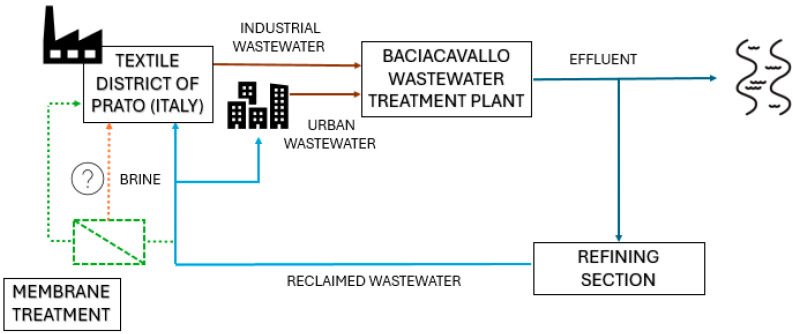
Current water cycle in the textile district of Prato (continuous lines) and proposed solution (dashed lines).

The textile district of Prato (Italy) is home to the largest water recycling plant in Europe. This plant enables the wastewater treatment plant’s (WWTP) effluent to be partially reused for industrial and municipal purposes, thereby closing the water cycle and promoting sustainable resource use (Figure 1). The refining plant is located downstream of the Baciacavallo WWTP, a facility managed by the company G.I.D.A. S.p.A. (hereafter G.I.D.A.) which treats both industrial and urban wastewater [5].

The reclaimed wastewater from the refining section is currently characterized by high hardness (32 °F on average), which is a crucial parameter in textile finishing processes. For instance, water hardness can cause dye precipitation and promote dye aggregation, resulting in a loss of color and faulty dyeings [6]. According to Vajnhandl and Valh (2014) [2], the hardness level of moderate–high-quality water that can be used in finishing processes should be lower than 10 °F. For this reason, most of the textile companies apply ion exchange resin (IER) technology for water softening. Moreover, even textile companies that are not connected to the industrial aqueduct are forced to soften groundwater because of the high background hardness.

Ion exchange is defined as a reversible physical–chemical process that involves the removal of dissolved compounds by replacing ions from a liquid phase to a solid phase and vice versa [7]. Ion exchange resins are composed of a polymer matrix to which active functional groups are bonded by covalent bonds. Mobile ions, which may have positive (cationic resins) or negative (anionic resins) surface charges, are weakly bonded to these via electrostatic bonds. Ions of the same sign present in the solution to be treated can be exchanged with mobile ions of the same sign. In water softening applications, cationic resins are used to replace Ca^2*+*^ and Mg^2*+*^ ions found in hard water with Na^+^ ions, based on the following removal mechanisms.

$$2R^{-}{Na}^{+}+{Ca}^{2+}⟷R_{2}^{-}{Ca}^{2+}+2{Na}^{+}$$

$$2R^{-}{Na}^{+}+{Mg}^{2+}⟷R_{2}^{-}{Mg}^{2+}+2{Na}^{+}$$

where *R^−^* is the polymer matrix with the active functional group.

During the ion exchange process, there is an observed increase in the concentration of calcium and magnesium ions in the effluent up to a maximum acceptable concentration, known as the breakthrough concentration. Thereafter, the ion exchange process is interrupted to initiate the resin regeneration phase. During this phase, a backwash is necessary to mobilize the resin, eliminate any preferential channels formed during operation, and remove accumulated particles. Subsequently, the resin undergoes regeneration through the application of high-concentrated NaCl solutions (8–12% [8]). Following this, a series of rinses are conducted to ensure the removal of residual NaCl solutions from the resin [9].

The regeneration of the resins produces a wastewater stream containing sodium, chloride, and bivalent ions [10]. Since chlorides cannot be removed through the WWTP and the refining section, reclaimed wastewater is characterized by high chloride concentration, exceeding the limit set by Italian Law (Ministerial Decree No. 185/2003 [11]) for reclaimed wastewater reuse for irrigation (250 mg·L^−1^).

Membrane filtration represents a well-established technology for the purpose of water softening and, in the literature, there are many examples of nanofiltration (NF) application. The efficacy of this process in removing hardness is contingent upon several factors, including the quality of the water to be treated, the molecular weight cut-off (MWCO) of the membrane, and the transmembrane pressure (TMP). Schaep et al. (1998) [12] investigated the use of NF for the softening of groundwater, achieving a calcium rejection rate of 96% at a TMP of 10 bar. Orecki et al. (2004) [13] investigated the efficacy of NF for the treatment of surface water, demonstrating a reduction in the total water hardness by nearly 85% at 15 bar TMP. Izadpanah and Javidnia (2012) [14] demonstrated that NF enables the softening of diluted seawater with an efficiency of 96–98%, depending on the TMP, which ranged between 4 and 8 bar. Elazhar et al. (2021) [15] implemented a system comprising nanofiltration and reverse osmosis (RO) in series for the purpose of brackish water softening. The system was observed to reduce the total hardness with an efficiency exceeding 90% at 10 bar TMP, utilizing an NF membrane with an MWCO of 90 Da. In an experimental study conducted by Wang et al. (2020) [16], an NF system was tested on raw water taken from the Yellow River Reservoir, achieving a total hardness removal efficiency of 98% with a TMP of approximately 2 bar. In a further study, Ketharani et al. (2022) [17] evaluated the performance of a number of existing NF plants in the North Central Province of Sri Lanka. The findings indicated that the plants demonstrated an efficiency of over 80% in the removal of total groundwater hardness, operating at a TMP of 5 bar. In a recent study, Chen et al. (2023) [18] examined the efficacy of a UF + NF system in the advanced treatment of drinking water. The performance of three commercial NF membranes was evaluated, with a hardness removal efficiency in the range of 40-73% being observed, contingent on the specific membrane type.

One of the primary disadvantages of employing pressure-driven membrane processes, including NF, is the generation of the concentrate (or retentate) stream. The composition of the concentrate is analogous to that of the feed composition, exhibiting elevated concentrations of components that have been rejected by the membrane [19]. Regarding retentate treatment, the system known as zero liquid discharge (ZLD) is considered one of the most promising alternatives to the disposal of retentate, since the treatment results in a reduction in environmental pollution, a minimization of waste volume, and the production of freshwater with high recovery. The first stage of ZLD systems is usually composed of membrane-based technologies to increase water recovery and minimize the brine’s volume [20]. Therefore, NF represents a membrane-based technology that is specifically employed for retentate pretreatment due to its reliability and the absence of sludge production. However, its adoption is currently limited to low-salinity brines (Total Dissolved Solids (TDS) < 55,000 mg·L^−1^) to minimize fouling concerns [20,21].

To the best of the authors’ knowledge, there is a lack of studies in the existing literature regarding the application of membrane filtration to enhance the quality of reclaimed wastewater and treat the produced retentate.

The principal novelty of this work is the study of an industrial-scale pilot plant of ultrafiltration and nanofiltration in series (UF-NF) for the removal of hardness from the reclaimed wastewater produced from the refining plant situated downstream of the WWTP of the textile district of Prato. Moreover, an NF stage comprising two NF membranes in series was applied to treat the byproduct of the UF-NF system.

The main objective is to produce water with hardness characteristics suitable for direct reuse in wet textile finishing processes, particularly dyeing, thereby increasing the potential for greater utilization of the industrial aqueduct. This will facilitate the substitution of ion exchange and the induction of chloride reduction in the Prato wastewater treatment system. Furthermore, the treatment of the produced retentate aims to reduce the volume of the concentrate and increase the water recovery. To this end, a pilot plant comprising ultrafiltration and nanofiltration was installed downstream of the refining section of the WWTP and operated for a period of five months. The performance of two distinct types of NF membranes was evaluated, and the impact of permeate flux on the overall efficiency of the system was assessed.

This article is a revised and expanded version of a paper entitled “Membrane treatment for water recycling and resource recovery in a textile district” [22], which was presented at the seventh MEMTEK International Symposium on Membrane Technologies and Applications, Istanbul (Turkey), 17–19 October 2023.

## 2. Materials and Methods

### 2.1. Water Characteristics

The feed stream of the UF-NF system was the reclaimed wastewater from the refining plant located downstream of the Baciacavallo WWTP, situated within the textile district of Prato, Italy. The influent wastewater is treated with a conventional activated sludge process followed by coagulation–flocculation treatment and ozonation [5]. The refining plant consists of a decoloration and flocculation stage followed by sand filtration and filtration on activated carbon. The main characteristics of the effluent from the refining plant are reported in Table 1.

### 2.2. Experimental Setup

**Figure 2:**
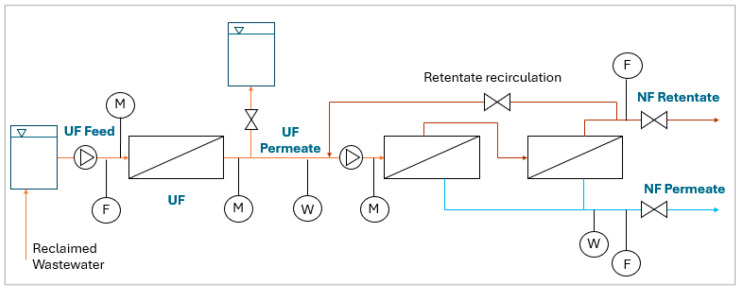
UF-NF pilot plant scheme (F: flowmeter; M: manometer; W: water meter).

The plant was designed by the company Fildrop S.r.l. (Prato, Italy) and installed downstream of the refining plant from July to December 2022. The scheme of the pilot plant is reported in Figure 2.

The reclaimed wastewater went to a feed tank, where it was stored as the influent of the pilot plant. The water was then pumped (model CEA 120/5/C, Lowara Srl Unipersonale, Xilem Inc., Washington, DC, USA) to the first section of the plant, composed of a UF hollow fiber membrane module of 20 nm pore size (dizzer XL 1.5 MB 25W, Inge GmbH, Greifenberg, Germany) with an effective membrane area of 25 m^2^. The ultrafiltration process was carried out in a dead-end mode at a constant flow rate of 2 m^3^·h^−1^ (i.e., 80 L·m^−2^∙h^−1^), measured with a flowmeter. The inlet pressure was kept constant at almost 2 bar. The TMP was monitored by measuring the inlet and outlet pressures with a manometer. When the TMP exceeded 1 bar, it was necessary to perform membrane cleaning. First, membrane flushing was carried out for about 1 min. During this phase, permeate production was halted, and the feed water that remained in the membrane was completely removed and discharged into the sewer system. Subsequently, membrane backwashing was carried out: this involved pumping part of the produced permeate (stored in a dedicated tank) in the opposite direction to the operating flow at a pressure of approximately 2 bar for about 2 min. The purpose of this process was to remove the cake layer from the membrane, thereby reducing filtration resistance. At the end of the backwashing process, the water was discharged into the sewer system.

The UF permeate not stored for backwashing was pumped (model CEA 120/5/C, Lowara Srl Unipersonale, Xilem Inc., Washington, DC, USA) to the subsequent NF process, composed of two NF spiral-wound membrane modules installed in series. The NF process was carried out in a cross-flow mode with a constant exercise pressure of 9 bar, monitored by a manometer. Two types of membranes were tested (NANO9-2540 and NANO7-2540, Oltremare S.p.A., Mann+Hummel Group, Ludwigsburg, Germany) with membrane areas of 2.6 m^2^ each, characterized by different chloride rejections. The main characteristics of the membranes tested are reported in Table 2.

The membrane NANO9 was tested with three different permeate flow rates, 200-180-140 L·h^−1^ (i.e., 38-35-27 L·m^−2^∙h^−1^), with a constant concentrate production of 80 L·h^−1^, measured with two flowmeters.

Finally, the produced permeate was discharged into the sewer system while the retentate was partly recirculated at the head of the NF process to adjust the permeate production [14], and partly discharged.

The plant operated five days per week, for eight hours per day. During the operational period of the plant, parameters such as the volume of water entering the NF section, the volume of produced permeate, the TMP of the UF, the NF exercise pressure, and the electric energy (EE) required by the pumps upstream of the UF and NF stages were measured and registered hourly.

During the experiment, four water samples were taken twice per day from the UF feed, UF permeate, NF permeate, and NF retentate to analyze parameters, such as conductivity, temperature, total hardness, chlorides, and turbidity. In addition, the Silt Density Index (SDI) of the feed stream was determined according to the test procedure described by Fritzmann et al. (2007) [23]. The SDI is used to estimate the fouling potential caused by fine suspended organic or inorganic colloids, and a pretreatment upstream of the NF is strongly recommended if the value exceeds 4 [23].

### 2.3. Sample Analysis Procedure

Conductivity and temperature were measured using the instrument XS™ COND7 Vio (Carpi, Italy).

In order to determine the turbidity, samples were homogenized with a Miccra™ MiniBatch D9 homogenizer (Buggingen, Germany). Turbidity was determined with a HACH™ TL2310 LED (Loveland, CO, USA) benchtop turbidimeter, calibrated with the StablCal™ Calibration Set (0–4000 NTU) (Hach Company, Loveland, CO, USA) and subsequently validated with the GELEX™ Secondary Turbidity Standard Kit (0–4000 NTU) (Hach Company, Loveland, CO, USA) .

The chloride ions underwent a reaction with mercury (II) thiocyanate, resulting in the formation of weakly dissociated mercury (II) chloride. The liberated thiocyanate formed, with the iron (III) ions, the red iron (III) thiocyanate, which was determined photometrically at a wavelength of 480 nm.

Finally, the total hardness of the water samples was determined by employing a magnesium EDTA complex, which exchanges with calcium. The released magnesium forms a red/violet complex with calmagite at a pH of 10. The absorbance of the complex was quantified at 620 nm, and the result is expressed in milligrams per liter of calcium carbonate.

### 2.4. Retentate Treatment

The retentate quantity is a function of the water recovery rate (*R*), expressed as the percentage of the volume of freshwater produced (*Q_p_*) to the total volume of feed water (*Q_f_*) [20].

(1) $$R=\frac{Q_{p}}{Q_{f}}\times 100$$

The volume of retentate produced (*Q_c_*) can be determined by the following equation.

(2) $$Q_{c}=Q_{f}-Q_{p}$$

**Figure 3:**
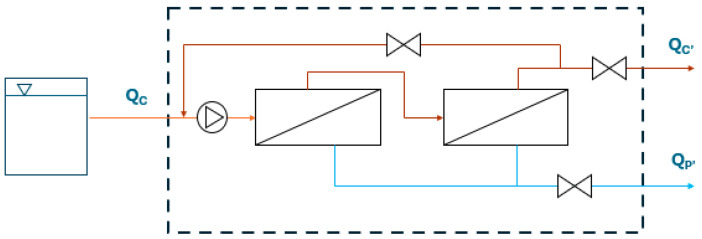
NF scheme for the retentate treatment.

During the experiment, a test for retentate treatment was conducted. The concentrate was collected in a tank with a volume of 500 L. It was then treated through the two NF membranes connected in series, as detailed in Section 2.2. Consequently, the NF apparatus was disconnected from the UF section and connected to the tank. The scheme of the apparatus is reported in Figure 3.

The experiment was conducted by testing the NANO7 membranes and maintaining constant a permeate flux of 21 L·m^−2^·h^−1^. The test was conducted over a three-hour period, during which water samples were taken three times from the NF inlet, NF permeate, and NF concentrate. The following parameters were analyzed: conductivity, temperature, hardness, chlorides, and turbidity. Additionally, the electric energy required by the pump was monitored.

## 3. Results and Discussion

### 3.1. System Rejection Analysis

The principal characteristics of the inflows and outflows with reference to the period during which the experiment was conducted (from July to December 2022) are reported in Table 3. It can be observed that the feed stream was characterized by high hardness (26.0 ± 4.0 °F) and high chloride concentration (740 ± 166 mg·L^−1^), exceeding the limit set by Italian Law (Ministerial Decree No. 185/2003 [11]) for wastewater reuse for irrigation and civil purposes (250 mg·L^−1^). Regarding the SDI, the reclaimed wastewater showed an average value of 5.15 ± 0.60. According to Fritzmann et al. (2007) [23], the UF stage upstream of the NF was strongly recommended and, therefore, installed. UF membranes are, in fact, porous membranes, characterized by a large pore size (1–20 nm). They are highly effective at trapping macromolecules such as suspended matter, colloids, particles, viruses, and bacteria, preventing NF membrane fouling [18].

**Figure 4:**
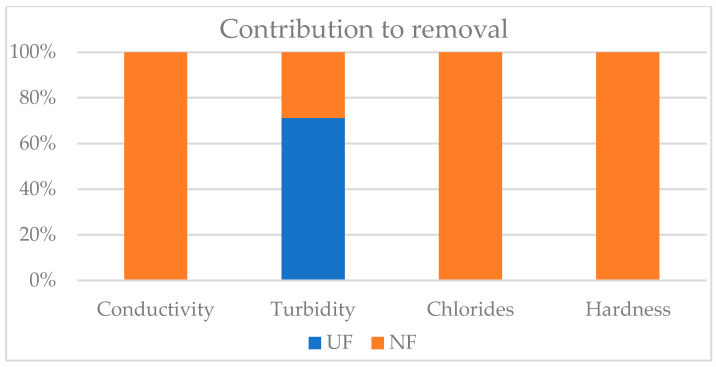
Contribution of UF and NF stages to the removal efficiency of conductivity, turbidity, chlorides, and hardness.

The whole UF-NF system reduced conductivity, turbidity, hardness, and chlorides by, on average, 89%, 61%, 98%, and 91%, respectively. According to Vajnhandl and Valh (2014) [2], the obtained NF permeate can be considered high-quality water (hardness < 9 °F and Cl^−^< 500 mg·L^−1^) suitable for all textile finishing processes. The graph reported in Figure 4 presents the contribution of UF and NF to removal efficiency of conductivity, turbidity, chlorides, and hardness.

As already highlighted, the UF step was necessary due to the measured SDI for the feed of the pilot plant. It is recommended as a pretreatment to avoid the fouling of the NF membranes and damage to the equipment [24,25]. Nevertheless, the reduction efficiencies of the UF stage are negligible, except for turbidity values, whose contribution to rejection represents approximately 70% of the overall turbidity rejection of the UF-NF system. This is primarily due to the fact that the UF membrane filtration mechanism is predominantly governed by mechanical screening, which exhibits a limited capacity to intercept pollutants that are smaller than its pore size. Consequently, the removal effect on soluble and inorganic pollutants in water is minimal [16].

The mean rejection rates for the NF stage, expressed in relation to the UF permeate, are 89 ± 4%, 33 ± 35%, 98 ± 1%, and 91 ± 4% for conductivity, turbidity, hardness, and chlorides, respectively. The performance of nanofiltration is influenced by several factors, including the parameters of pressure, temperature, recovery, and the characteristics of the feed water [26]. As a result, the aforementioned results are not always in accordance with those reported in the existing literature. Orecki et al. (2004) [13] employed an NF tubular module to treat surface water with a TMP of 15 bar. The system was observed to remove conductivity, turbidity, and chlorides with efficiencies of approximately 68%, 86%, and 85%, respectively. As stated by Izadpanah and Javidnia (2012) [14], an NF spiral-wound membrane can reject hardness, salinity, and conductivity from seawater with an efficiency within the ranges of 96–98%, 77–86%, and 79–89%, respectively, depending on the TMP applied. Elazhar et al. (2021) [15] demonstrated that NF can reject conductivity, chlorides, and total hardness by more than 95% from brackish water at a TMP of 10 bar. Wang et al. (2020) [16] observed that NF exhibited a notable removal effect on total hardness, conductivity, and chlorides, with respective efficiencies of 98%, 90%, and 81%, at a TMP of 2.22 bar. Chen et al. (2023) [18] employed a UF-NF system to improve the quality of drinking water. The researchers discovered that UF is primarily effective in removing turbidity, with an average rejection of almost 88.6%. NF demonstrated the ability to remove conductivity and hardness by 25–29% and 40–73%, respectively, depending on the specific membrane type and the system’s recovery. In a further study, Ketharani et al. (2022) [17] applied three types of NF membranes to soft groundwater. The findings indicated that, with a TMP of 5 bar, the system was capable of removing Cl^-^ and conductivity by 83% and 96% on average, respectively, and hardness by 60–98%. The efficacy of the system was contingent upon the specific membrane type.

In this study, the performance of the nanofiltration section in terms of rejection and energy efficiency was evaluated by comparing two different types of membranes and different permeate fluxes. The results are presented in the following paragraphs.

### 3.2. Pilot Plant Study with Different Types of NF Membranes

NANO7 and NANO9 membranes were tested with a constant permeate flow rate of 140 L·h^−1^, corresponding to a permeate flux of 27 L·m^−2^·h^−1^. The results are reported in Table 4.

Comparing the two membranes from the permeate characteristics perspective, both can produce high-quality water in terms of hardness (<9 °F) and chloride concentration (<500 mg·L^−1^), suitable for all textile finishing processes [2].

As evidenced in Table 2, both membranes demonstrate high efficiency in rejecting MgSO₄ (which contributes to water hardness), exceeding 97%. However, they exhibit differential performance in NaCl rejection, with membrane NANO9 characterized by a lower MWCO.

**Figure 5:**
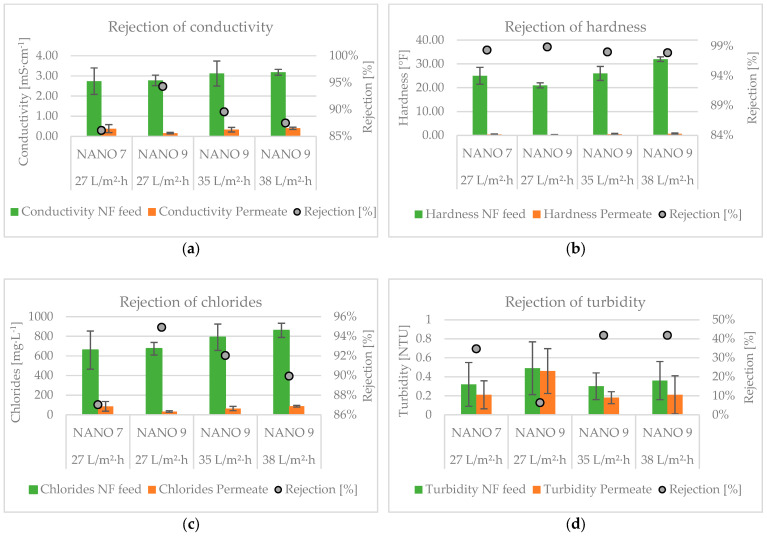
Conductivity (**a**), hardness (**b**), chlorides (**c**), and turbidity (**d**) in the inlet and the permeate of the NF section.

The membrane NANO7 exhibits superior turbidity rejection, although the rejection rate does not exceed 35%. This outcome is incongruent with the expected results. Indeed, higher MWCOs should result in lower turbidity rejection due to the passage of small molecules [27]. In this case, the elevated turbidity present in the feed stream of the membrane NANO9, despite its lower MWCO, is likely to diminish the removal efficiency in comparison with the membrane NANO7 (Figure 5).

Regarding total hardness removal, both membranes NANO7 and NANO9 exhibit excellent rejection rates (98% and 99%, respectively), resulting in permeates with total hardnesses of 0.41 ± 0.18 °F and 0.24 ± 0.11 °F, respectively. Membrane NANO9 exhibits superior performance in the removal of chlorides and conductivity, with respective efficiencies of 95% and 94%. However, the permeate produced by membrane NANO7 is distinguished by a chloride concentration of 86 ± 49 mg·L^−1^, which is below the limit set by Italian Law for reclaimed water reuse for irrigation purposes (250 mg·L^−1^).

The results clearly demonstrate that both membranes are effective in hardness reduction and chloride rejection. In contrast to the removal of turbidity, the rejection of chlorides and conductivity is consistent with the different MWCOs of the membranes. The influent concentrations do not exert an influence on these processes, as they are found to be very similar to each other (Figure 5). Finally, in regard to the issue of hardness removal, it is observed that neither MWCO nor the hardness of the feed water (Figure 5) exhibit a significant impact on this process.

### 3.3. Pilot Plant Study with Different Permeate Fluxes

During the experiment, three permeate flow rates were tested with the membrane NANO9. The obtained results are reported in Table 4. It can be observed that the hardness rejection is higher than 98% and it does not depend on the permeate flux, always obtaining values lower than 1 °F. On the other side, turbidity removal increases with the permeate production, but it does not exceed 42%. Regarding conductivity and chloride rejections, the results show that they are higher for lower permeate flux. In particular, conductivity removal increases from 87% to 94% and chloride rejection increases from 90% to 95%, decreasing the permeate flux from 38 to 27 L·m^−2^·h^−1^.

In order to provide an explanation for this behavior, it is necessary to observe that the fluid entering the NF exhibits time-varying quality characteristics. In a recent study conducted by Qadir et al. (2023) [28], a decrease in salt rejection was observed as the feed concentration increases. As illustrated in Figure 5, conductivity, hardness, and chloride content were observed to be higher at higher permeate fluxes. These two variables (feed flow concentration and permeate flow) exert an influence on the phenomenon of concentration polarization. This phenomenon is defined as the increase in the solute concentration over the bulk feed solution, occurring in a thin boundary layer at the feed side of the membrane surface [26]. In particular, an elevated feed concentration and a higher permeate flux facilitate the advancement of this phenomenon, resulting in a decline in rejection [23,29].

Regarding turbidity, it was observed that the characteristics of the inlet fluid exerted a significant influence on the degree of rejection. It is evident that the rejection remains unchanged when the permeate flow is 38 and 35 L·m^−2^·h^−1^, as the inlet turbidity is comparable (0.36 ± 0.20 vs. 0.30 ± 0.14 NTU, respectively). Conversely, a decline in rejection is observed with a permeate flux of 27 L·m^−2^·h^−1^, when the inlet turbidity exceeds that of the preceding cases (0.49 ± 0.28 NTU).

### 3.4. Retentate Treatment

The recoveries of the NF system for different experimental conditions are reported in Table 5.

The retentate treatment experiment was carried out by using the concentrate produced with the NANO7 membrane with 27 L·m^−2^·h^−1^ of permeate flux. The recovery of the NF system with these operating conditions is 69% (Table 5).

The results of the application of the NF system for the retentate treatment are reported in Table 6. Regarding conductivity, turbidity, chlorides, and hardness, the removal efficiencies are, on average, 89%, 71%, 91%, and 98%, respectively.

**Figure 6:**
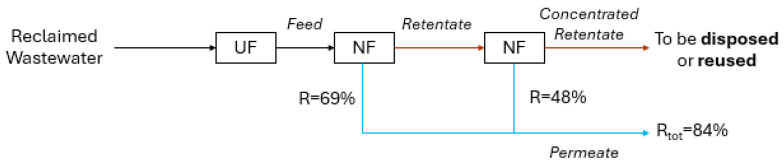
Overall recovery of the system composed of the UF-NF plant and retentate treatment section.

The retentate treatment through NF allows recovery of almost 48% of the permeate (Figure 6). This result is in accordance with the existing literature. According to Panagopoulos et al. (2019) [20], the application of reverse osmosis (RO) for brine’s treatment leads to a maximum water recovery of 50% if the inlet TDS is lower than 70,000 mg·L^−1^. In another study, Sudhakar et al. (2016) [30] investigated the sequential operation of an industrial-scale integrated membrane process (RO and UF) to achieve high permeate recovery in a textile dyeing water resource recovery facility. They found that the treatment of primary RO brine by secondary RO was characterized by a 50% recovery.

In conclusion, the application of a two-stage NF membrane system resulted in a 15% increase in permeate production, thereby achieving an overall recovery rate of 84%. The remaining concentrated retentate represents 16% of the total feed stream entering the NF system, and it is characterized by Cl^−^ equal to 3835 ± 1073 mg·L^−1^ and hardness of 149 ± 43 °F (Table 6). Rather than disposing of them, it would be more beneficial to recover the value-added compounds within the textile processes to achieve zero liquid discharge [1].

According to the existing literature [20], membrane-based technologies (e.g., reverse osmosis, forward osmosis, membrane distillation, membrane crystallization, and electrodialysis) and thermal-based technologies (e.g., brine concentrators and crystallizers, multi-stage flash distillation, multi-effect distillation, spray dryers, eutectic freeze crystallization, and wind-aided intensified evaporation) can be employed to produce freshwater and high-purity salts suitable for reuse [31].

In a recent work, Partal et al. (2022) [32] studied a pilot-scale system equipped with ozone oxidation, NF, RO, and IER units to treat the RO brine derived from textile wastewater treatment. This system successfully recovered both water and salt solutions, which were subsequently reused in textile production processes, such as polyester yarn and cotton home textile manufacturing.

In the specific case of Prato, not all the water exiting the refining plant requires softening. The concentrated retentate could potentially be diluted with non-softened water, allowing its reuse in processes that do not demand high-quality water, such as washing-off stages and equipment cleaning [2]. However, these aspects have not yet been investigated and therefore merit further study.

### 3.5. Electricity Demand

**Figure 7:**
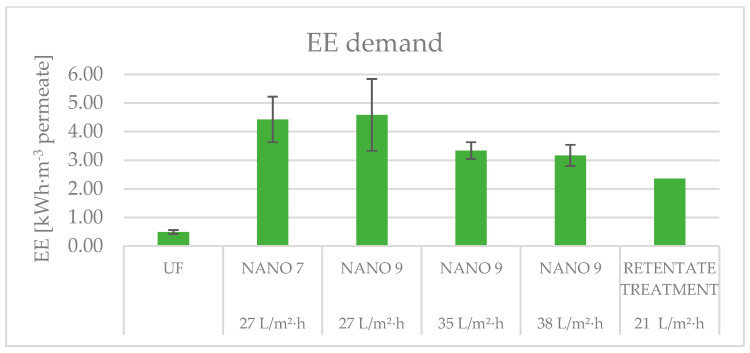
Electric energy demand for each section of the system.

The graph reported in Figure 7 shows the electricity consumed by each section of the system. As can be observed, ultrafiltration is characterized by an average EE consumption of 0.49 ± 0.07 kWh·m^−3^ of produced permeate while the greatest amount of energy is required for nanofiltration. It varies from 3.17 ± 0.37 to 4.58 ± 1.26 kWh·m^−3^ of permeate, depending on the type of membrane, the permeate flow rate, and the characteristics of the feed.

The membrane NANO7 exhibits a lower EE consumption rate (4.42 kWh per cubic meter of permeate) compared to the membrane NANO9 (4.58 kWh per cubic meter of permeate). This is primarily attributed to the membrane NANO7’s higher MWCO.

The findings pertaining to the membrane NANO9 indicate that elevated EE consumption is associated with reduced permeate fluxes. This outcome is a direct result of the reduction in permeate flux observed with the increased recirculation of the NF concentrate. In the pilot plant used in this study, permeate flux was regulated by adjusting the recirculation of the concentrate. The pump, which served both the nanofiltration feed and concentrate recirculation, lacked an inverter, preventing adjustment of the motor speed. As a result, the motor speed remained constant, leading to higher energy consumption. In a future scale-up of the plant, energy consumption could be optimized by using pumps equipped with inverters that can adapt to varying operating conditions.

Furthermore, the elevated energy expenditure is attributable to the reduced temperature of the water, as illustrated in Table 4. Indeed, a reduction in temperature results in an increase in water viscosity, which consequently leads to an elevation in TMP [18]. Consequently, to maintain the same operating pressure, the system requires greater electrical input. In a future scale-up of the plant, measures should be implemented to keep the process water temperature at a constant level. The pilot plant in the present study was installed in an outdoor environment, directly exposed to atmospheric temperature variations. To reduce temperature fluctuations, the plant should be moved to a controlled environment, equipped with aeration systems to maintain a constant temperature.

Another crucial element to consider is the issue of membrane fouling. Indeed, the experiment began with a permeate flux of 38 L·m^−2^·h^−1^, which was subsequently modified to 35 L·m^−2^·h^−1^ and then decreased to 27 L·m^−2^·h^−1^, without a prior membrane cleaning procedure [33]. It is possible that membrane fouling may have influenced the EE consumption, as it causes permeability reduction, which in turn results in a considerable energy demand [34]. To minimize energy expenditure associated with membrane fouling, it is necessary to optimize system operating conditions in addition to implementing pretreatment systems. For instance, increasing the cross-flow velocity and decreasing the system recovery can lead to a reduction in concentration polarization, which in turn decreases the risk of scaling [26]. Furthermore, parameters such as pH and temperature of feed should be monitored to prevent membrane fouling [33].

Ultimately, the EE necessary for retentate treatment is approximately 2.4 kWh·m^−3^ of permeate generated by the UF-NF system, in alignment with the findings of Panagopoulos and Haralambous (2020) [35]. These researchers asserted that the specific energy consumption of RO and NF systems for brine treatment falls within the range of 2–6 kWh·m^−3^ of permeate produced.

The consumption of electricity is directly proportional to the osmotic pressure of the feed stream [36]. It is expected that the UF-NF system will be installed upstream of a single (or clustered) textile company, resulting in the production of soft water and reduction of chlorides throughout the wastewater treatment system of the district. A reduction in chloride concentration will result in a corresponding reduction in the osmotic pressure of the water that must be softened, thus lowering the operating pressures of the membranes and reducing electricity consumption. Furthermore, the installation of the plant along the industrial aqueduct line will allow the water pressure in the distribution line (approximately 4 bar) to be exploited, thereby reducing energy consumption.

## 4. Conclusions

The experimental results reported in this work suggest that the UF-NF system is promising to be used as a post-treatment step to make reclaimed wastewater suitable for all textile finishing processes and to widen the opportunity for reuse (e.g., urban irrigation). It could be an alternative process to ion exchange resins, promoting water recycling and resource recovery and preventing the addition of further salt load.

In this study, the contribution of UF to rejections was found to be negligible, except for turbidity. Indeed, its contribution to rejection represents approximately 70% of the overall turbidity rejection of the UF-NF system. This result confirms the importance of applying UF to prevent NF fouling.

Two NF membranes were tested. It was observed that changing membranes does not affect hardness removal, which is higher than 98%, obtaining a permeate characterized by a total hardness lower than 1 °F. The lower MWCO of membrane NANO9 results in higher conductivity and chloride rejections compared to membrane NANO7, demonstrating an excellent efficiency that exceeds 94%. Nevertheless, membrane NANO7 produces a permeate characterized by Cl^−^ equal to 86 ± 49 mg·L^−1^ and requires less EE consumption with the same operating conditions. With regard to the process of turbidity removal, the higher turbidity in the feed stream of the membrane NANO9, despite its lower MWCO, is likely to reduce its removal efficiency compared to the membrane NANO7.

Membrane NANO9 was tested with different permeate flow rates. Hardness removal does not depend on permeate production, exhibiting efficiencies higher than 98%. Chlorides and conductivity rejections were found to be better for lower permeate fluxes. The observed result can be attributed to the phenomenon of concentration polarization, which is facilitated by an elevated feed concentration and a higher permeate flux, resulting in a decline in rejection.

Regarding EE demand, it is higher with reduced permeate fluxes. This result is primarily due to the increased recirculation of the retentate. Then, the elevated energy expenditure is attributable to the reduced temperature of the water, which corresponds to a higher water dynamic viscosity, leading to a greater electrical input. Finally, membrane fouling may have influenced the EE consumption, as it causes membrane permeability reduction. In a future scale-up of the plant, EE consumption could be optimized by the following measures: (I) the employment of pumps equipped with inverters capable of adapting to variable operating conditions; (II) the implementation of measures to keep the process water temperature at a constant level; and (III) the optimization of system operating conditions (i.e., cross-flow velocity and recovery) and the monitoring of the pH and temperature of the feed to control membrane fouling.

Ultimately, during the experiment, a test for retentate treatment was carried out. Two NF stages were applied to reduce the retentate volume and increase the recovery of the UF-NF system. The application of a two-stage NF membrane system results in a 15% increase in permeate production, thereby achieving an overall recovery rate of 84%. It can be considered a reliable pretreatment that should be followed by other processes to achieve zero liquid discharge. Rather than disposing of the remaining concentrated retentate, it would be more beneficial to recover the value-added compounds within the textile processes by the application of membrane-based and thermal-based technologies. Considering that not all the water exiting the refining plant of Prato requires softening, the concentrated retentate could potentially be diluted with non-softened water, allowing its reuse in processes with lower water quality requirements.

Future studies should focus on the NF retentate treatment and its potential reuse applications. Furthermore, a mathematical model should be developed to represent the chloride cycle in the Prato wastewater treatment system. This model would enable the monitoring of chloride concentration in the effluent from the refining plant and the evaluation of the effectiveness of the installation of UF-NF plants upstream of the textile mills in reducing chloride concentration in the system. This would permit the water to be reused for other purposes (e.g., irrigation) and reduce the osmotic pressure of the water to be treated, thereby reducing the costs associated with EE consumption. Finally, an economic feasibility study of the system should be conducted to assess Capex and Opex costs and to evaluate the economic viability of full-scale plants.

In conclusion, the UF-NF pilot plant was tested in the specific context of the Prato textile district, which is characterized by a centralized wastewater treatment system. It is important to note that companies in the district do not soften all the water, but only a portion (e.g., the water used for textile dyeing). Consequently, in the event of implementing a full-scale UF-NF system, instead of a single large plant, it will be necessary to design multiple small-scale plants located at the point of use in each factory to meet the specific needs of the companies. This consideration implies that the system studied in this work can also be applied in situations where a decentralized wastewater treatment system is in place, with each company having its own WWTP. However, it is essential to conduct pilot plant studies to assess the impact of water quality on system efficiency.

## Figures and Tables

**Table 1:** Average annual values of the main characteristics of the effluent from the refining plant, related to the year 2022 (mean ± STD).

Parameter	Unit	Refining Plant
pH	-	7.54 ± 0.16
TSS	mg·L^−1^	1.01 ± 0.11
Hardness	°F	31.6 ± 4.9
Chlorides	mg·L^−1^	702 ± 155
Turbidity	NTU	0.53 ± 0.24
COD	mg·L^−1^	20.3 ± 5.43
N-NH_4_^+^	mg·L^−1^	2.82 ± 4.25
N-NO_2_^−^	mg·L^−1^	0.20 ± 0.22
N-NO_3_^−^	mg·L^−1^	12.5 ± 6.17
Absorbance (λ = 420 nm)	-	0.007 ± 0.006

**Table 2:** UF and NF modules’ characteristics.

Module Specification	Unit	UF Module	NF Module	
Reference		dizzer XL 1.5 MB 25W	NANO9 2540	NANO7 2540
Manufacturer		Inge GmbH	Oltremare S.p.A.	Oltremare S.p.A.
Active membrane area	m^2^	25	2.6	2.6
Membrane configuration		Hollow Fiber	Spiral-Wound	Spiral-Wound
MgSO_4_ rejection	%	-	>97 ^1^	>97 ^1^
NaCl rejection	%	-	89–95 ^1^	45–55 ^1^
Pore size	nm	20	-	-
Pressure max	bar	5	41	41
Temperature max	°C	40	45	45
pH range	-	1–13	3–10	3–10

^1^ Permeate flow and salt rejection based on the following test conditions: 2000 ppm MgSO_4_, 500 ppm NaCl, 25 °C, and 10% recovery at the pressure of 4.8 bar.

**Table 3:** UF feed and permeate and NF permeate and retentate values (mean ± STD).

Parameter	Unit	UF Feed	UF Permeate	NF Permeate	NF Retentate	System Rejection
SDI	-	5.15 ± 0.60	-	-	-	-
Conductivity	mS·cm^−1^	2.96 ± 0.59	2.96 ± 0.58	0.34 ± 0.15	5.93 ± 1.26	89 ± 4%
Temperature	°C	27.0 ± 6.1	27.0 ± 5.1	28.0 ± 5.3	28.0 ± 5.2	-
Hardness	°F	26.0 ± 4.0	26.0 ± 4.0	0.48 ± 0.25	84.0 ± 106	98 ± 1%
Chlorides	mg·L^−1^	740 ± 166	737 ± 166	72.0 ± 37.0	2126 ± 1768	91 ± 4%
Turbidity	NTU	0.65 ± 0.34	0.34 ± 0.20	0.22 ± 0.15	0.30 ± 0.14	61 ± 26%

**Table 4:** Effect of the permeate flow rate and the type of membrane on NF rejection (%), and effect of temperature on the water dynamic viscosity (mean ± STD).

Permeate Flux [L·m^−2^·h^−1^]	Membrane	Rejection [%]				T [°C]	Dynamic Viscosity [Pa·s]
		Conductivity	Hardness	Chlorides	Turbidity		
27	NANO 7	87 ± 6	98 ± 1	88 ± 6	33 ± 41	23 ± 5	9.32 × 10^−4^
27	NANO 9	94 ± 1	99 ± 1	95 ± 1	5 ± 6	25 ± 2	8.90 × 10^−4^
35	NANO 9	90 ± 2	98 ± 1	92 ± 2	38 ± 26	31 ± 2	7.81 × 10^−4^
38	NANO 9	87 ± 2	98 ± 1	90 ± 1	37 ± 56	33 ± 2	7.49× 10^−4^

**Table 5:** Recoveries of the NF system for different experimental conditions.

Flow Rates	U. o. M.*	NANO9 38 L·m^−2^·h^−1^	NANO9 35 L·m^−2^·h^−1^	NANO9 27 L·m^−2^·h^−1^	NANO7 27 L·m^−2^·h^−1^
Permeate	m^3^·h^−1^	0.19 ± 0.03	0.16 ± 0.04	0.12 ± 0.04	0.12 ± 0.02
Feed	m^3^·h^−1^	0.25 ± 0.02	0.22 ± 0.06	0.18 ± 0.05	0.17 ± 0.06
NF recovery	-	75 ± 8%	73 ± 3%	67 ± 15%	69 ± 10%

* U.o.M.: Unit of Measurement.

**Table 6:** Efficiency of the NF system for the retentate treatment (mean ± STD).

	Conductivity	Turbidity	Chlorides	Hardness
	mg·L^−1^	NTU	mg·L^−1^	°F
Retentate	6.11 ± 0.01	1.9 ± 1.6	1794 ± 17	69 ± 0.8
Permeate	0.66 ± 0.21	0.2 ± 0.1	163 ± 61	1.07 ± 0.21
Concentrated retentate	9.69 ± 1.94	15 ± 24	3835 ± 1073	149 ± 43
Rejection	89 ± 3%	71 ± 40%	91 ± 3%	98 ± 0.3%

## Data Availability

The original contributions presented in this study are included in the article. Further inquiries can be directed to the corresponding author.
